# An in-depth comparison of vascular inflammation on ultrasound, FDG-PET/CT and MRI in patients with suspected giant cell arteritis

**DOI:** 10.1007/s00259-025-07088-3

**Published:** 2025-02-04

**Authors:** Marieke van Nieuwland, Pieter H. Nienhuis, Cees Haagsma, Kornelis S. M. van der Geest, Nils R. L. Wagenaar, Auke P. A. Appelman, Onno D. Vijlbrief, Lenny van Bon, Edgar M. Colin, Elisabeth Brouwer, Riemer H. J. A. Slart, Celina Alves

**Affiliations:** 1 Department of Rheumatology and Clinical Immunology, University of Groningen, University Medical Center Groningen, Groningen, The Netherlands; 2 Department of Rheumatology and Clinical Immunology, Hospital Group Twente (Ziekenhuisgroep Twente), Almelo, the Netherlands; 3 Medical Imaging Center, Department of Nuclear Medicine and Molecular Imaging, University of Groningen, University Medical Center Groningen, Groningen, The Netherlands; 4 Department of Nuclear Medicine, Hospital Group Twente, Almelo, The Netherlands; 5 Medical Imaging Center, Department of Radiology, University of Groningen, University Medical Center Groningen, Groningen, The Netherlands; 6 Department of Radiology, Hospital Group Twente (Ziekenhuisgroep Twente), Almelo, the Netherlands; 7 Department of Rheumatology, Radboudumc, Nijmegen, The Netherlands; 8 Biomedical Photonic Imaging Group, Faculty of Science and Technology, University of Twente, Enschede, The Netherlands

**Keywords:** Giant Cell Arteritis, Imaging, Diagnosis, Ultrasound, PET, MRI

## Abstract

**Background:**

Giant cell arteritis (GCA) is a difficult to diagnose large vessel vasculitis. CDUS, FDG-PET/CT and MRI are increasingly used for GCA diagnosis. This study aims to assess vascular wall lesions in GCA suspected patients, directly comparing CDUS, FDG-PET/CT and MRI with each other.

**Methods:**

In a nested-case control study, consecutive GCA suspected patients were included. Scans were retrospectively assessed by two experts per imaging modality. Inter- and intraobserver agreement using Cohen’s or Fleiss Kappa were calculated to assess agreement between experts, a few duplicated scans and between imaging modalities. Sensitivity and specificity of the imaging modalities for overall diagnostic performance and for individual arteries were calculated.

**Results:**

In total, 42 patients were included. Overall diagnostic performance of imaging modalities was comparable. Sensitivity and specificity were highest in the temporal artery for CDUS (76% and 93%; Kappa > 0.7) and MRI (60% and 100%; Kappa > 0.7), and in the vertebral (61% and 100%; Kappa 0.56) and maxillary artery (52% and 100%; Kappa 0.75) for FDG-PET/CT. Agreement between all modalities for a positive temporal artery was 0.76, but only 0.28 between CDUS and FDG-PET/CT. Agreement for the axillary artery was 0.7 between CDUS and FDG-PET/CT.

**Conclusion:**

The temporal artery can be assessed by CDUS and MRI with good sensitivity and high specificity, and the axillary artery by CDUS and FDG-PET/CT with high agreement between the two modalities. In addition, the vertebral and maxillary artery can be assessed by FDG-PET/CT with good sensitivity and specificity, however the vertebral artery had moderate interobserver agreement.

**Supplementary Information:**

The online version contains supplementary material available at 10.1007/s00259-025-07088-3.

## Introduction

Giant cell arteritis (GCA) is a large vessel vasculitis that can lead to severe complications such as irreversible blindness, which can be prevented by adequate and timely treatment [1, 2]. Nonspecific symptoms often provide a diagnostic challenge. A temporal artery biopsy (TAB) is traditionally considered the gold standard for GCA diagnosis [3]. However, TAB has a reported low sensitivity with high heterogeneity between studies and moderate interobserver agreement, is invasive and results can take up to two weeks [3–5]. Therefore, imaging modalities for early GCA diagnosis are recommended for suspected GCA diagnosis as an alternative to histology [6, 7].

Colour duplex ultrasound (CDUS), Fluor-18-fluorodeoxyglucose Positron Emission Tomography combined with low dose Computed Tomography (FDG-PET/CT) and Magnetic Resonance Imaging (MRI) are increasingly used in GCA diagnosis [8, 9]. Following European Alliance of Associations for Rheumatology (EULAR) recommendations, CDUS of the axillary and temporal artery with its branches is recommended as a first diagnostic test [7]. Using CDUS, a 'halo sign’, which is a homogeneous, hypoechoic arterial wall thickening visible in longitudinal and transverse planes, is highly suggestive of GCA [10–12]. Alternatively, with whole-body FDG-PET/CT, increased glucose metabolism caused by inflammation of the arterial vessel wall can be visualized, including large vessels such as the aorta and its branches [13–15]. With contrast-enhanced cranial MRI, mural enhancement indicating presence of vasculitis of superficial and deeper vessels of the neck and head area can be visualized and form an alternative especially on suspicion of cranial involvement [16, 17].

CDUS, FDG-PET/CT and MRI each have good diagnostic performance for GCA diagnosis [8, 9, 18, 19]. However, most likely, not all vessels can be reliably evaluated using each imaging modality. Therefore, it would be ideal if the selection of a diagnostic test could be tailored to the specific arteries affected in a patient to minimize the risk of misdiagnosis, even though it can be unclear beforehand which arteries will be involved [19]. It is important to assess which GCA related vascular wall lesions can be detected by different imaging modalities with good reliability and validity, to achieve better interpretation of imaging results. This exploratory study aims to assess diagnostic performance and inter- and intraobserver agreement of vascular wall inflammation in individual arteries of GCA suspected patients, directly comparing CDUS, FDG-PET/CT and MRI with each other.

## Methods

### Study design

Patients with suspected GCA were recruited from the Hospital Group Twente GCA early in Twente (ZGT GET) prospective cohort for a nested-case control study, as described before [18]. All patients underwent CDUS, FDG-PET/CT and MRI for the original pilot study, that investigated diagnostic performance of the imaging modalities in GCA suspected patients as performed for general practice [18]. This study adds to existing knowledge by evaluating individual vascular wall lesions for each imaging modality after detailed re-assessment by two independent experts. This study was approved by the medical ethical committee and was performed in accordance with the declaration of Helsinki.

### Imaging and assessment

For the purpose of this study, CDUS, FDG-PET/CT and MRI images obtained for the original study were retrospectively re-assessed by two independent experts for each imaging modality. Patients suspected of GCA but not diagnosed with GCA by the expert panel were used as control group. Imaging data of 8 patients, including both GCA patients and patients ultimately not diagnosed with GCA, were duplicated to calculate intraobserver agreement. CH (~ 9 years of experience) and KvdG (~ 6 years of experience) were experts for CDUS assessment in GCA, RS (~ 15 years of experience of PET imaging in GCA) and NW (~ 15 years of experience of all-round PET imaging) for FDG-PET/CT and AA (> 10 years of experience in neuroradiology and PN (~ 2 years of experience) for MRI. The experts were blinded for all patient information, including final diagnosis, to avoid bias. For all imaging modalities, presence of GCA was determined by the experts for each assessable artery in addition to overall GCA diagnosis. After independent assessment, a consensus was reached between the experts in case of discrepancies. For more details regarding imaging modalities, see van Nieuwland et al., Supplementary File S1 [18; supplementary file S1]. In short, CDUS (Esaote MyLab Twice or Canon Aplio i800 ultrasound machine) of at least bilateral temporal (including its frontal and parietal branches) and axillary arteries was performed as part of standard diagnostic work-up in the pilot study. Stored images on the CDUS machine were used for retrospective re-assessment. Presence of a halo sign, compression sign and increased intima-media thickness (IMT) were assessed. Whole-body FDG-PET/CT (Siemens Biograph 40 mCT Flow, Siemens Healthineers, Knoxville, TN, USA) and 3 T MRI (Magnetom Skyra; Siemens Healthineers, Erlangen, Germany) of the head and neck area were performed for study purposes as early as possible, aimed within 3 to 5 working days after diagnostic work-up [18, 20]. For FDG-PET/CT, arterial inflammation of the head and neck area was retrospectively scored by the experts using a previously described score between 0 and 2 [20, 21]. Large vessel inflammation was scored based on a 0–3 score against liver uptake [13]. For MRI, the protocol outlined by Rhéaume et al. was followed [17]. Scans were retrospectively evaluated for GCA based on signal intensity and vessel wall thickening using a previously published scoring system between 0 and 3 [22].

### Data collection and case definition

Patient data were collected from electronic health records. Castor study management system (Ciwit B.V., The Netherlands, version 2020.2.24) was used for data management. The reference standard used in this study was the diagnosis after six months follow-up, blinded for imaging results. This reference standard was made by two independent experts (EC and KvdG) for the original pilot study [18]. The reference standard was not used for diagnosis, nor was it available to experts evaluating CDUS, FDG-PET/CT and MRI in the present study. In this study, the temporal, maxillary, auricular, occipital, facial, ophthalmic, carotid and vertebral arteries were considered cranial arteries.

### Statistical analysis

Mean values with standard deviation (SD) or median values with interquartile ranges (IQR) were used after testing for normality. Statistically significant differences (*p* < 0.05) between groups were assessed using an Independent Samples t-test, Mann–Whitney U test, Pearson Chi-Square or Fisher's Exact Test when appropriate. Inter- and intraobserver agreement were calculated using Cohen’s kappa adhering to the following terminology: no (≤ 0.00), slight (0.01–0.20), fair (0.21–0.40), moderate (0.41–0.60), substantial (0.61–0.80), almost perfect (0.81–0.99) and perfect agreement (1.00) [23]. Analyses involving assessments of individual arteries were performed with bilateral arteries combined. Hence in case of bilateral arteries, the total number of arteries was doubled.

#### Inter- and intraobserver agreement of vessel assessment

Inter- and intraobserver agreement were calculated for all assessable arteries on each imaging modality. Arteries that had no inter- and intraobserver agreement (Cohen’s kappa ≤ 0.00) after re-assessment were not deemed reliably assessable and excluded from further analyses (see Supplementary Table S1).

#### Overall inter-observer agreement and diagnostic performance of imaging modalities

A patient was considered positive for GCA on imaging if one or more of the reliably assessable arteries was scored positive for GCA. Using this, interobserver agreement for GCA diagnosis between the two experts per imaging modality was calculated. Using the reached consensus between the two experts, sensitivity and specificity of CDUS, FDG-PET/CT and MRI were calculated by cross-tabulation. The diagnosis after 6 months made by the expert panel was used as a reference diagnosis.

#### Diagnostic performance of individual vessels

In addition, sensitivity and specificity of each individual artery were calculated to determine which arteries have the optimal diagnostic performance on the different imaging modalities and show an in-depth comparison of vascular wall lesions on the three imaging modalities.

#### Overlap between imaging modalities

Finally, the agreement between different imaging modalities for each individual artery was determined using Cohen’s kappa (for 2 modalities) or Fleiss kappa (for 3 modalities). Comparative and agreement testing was paired and arteries with missing assessments were excluded from analysis.

## Results

### Baseline characteristics

In total, 42 patients were included. Mean age was 70.7 years (SD 7.7) and 26 patients were female. Of the total study population, 23 patients were diagnosed with GCA (GCA +) and 19 patients were suspected of GCA but ultimately not diagnosed with GCA (GCA-). Table 1A describes patient characteristics in more detail. For CDUS retrospective assessment, patients assessed with ESAOTE MyLab Twice CDUS were excluded for comparative and CDUS analyses (*n* = 9). These, CDUS images were static images saved on the US-machine and had a suboptimal quality for retrospective IMT measurements. Furthermore, CDUS images of *n* = 1 patient were not retrievable. Therefore, 32 patients (17 GCA +) were included for comparisons between all three imaging modalities and CDUS analyses. The selection of 8 duplicated patients was reduced to 6 in the CDUS group. Table 1B describes patient characteristics for the CDUS subgroup.

**Table 1:** Patient characteristics of the total study population

**A**	**GCA (*n* = 23)**	**No GCA (*n* = 19)**	**P-value**
Mean age in years (SD)	71.6 (6.9)	69.7 (8.7)	0.306
Female; % (n)	52.2	73.7	0.153
CRP (mg/L); median (IQR)	35.0 (9.5–68.3)	12.0 (2.0–27.0)	0.018
ESR (mm/hr); median (IQR)	52.0 (30.0–93.0)	27.0 (16.0–49.0)	0.012
Cranial symptoms^1^; % yes (n)	95.7 (22)	89.5 (17)	0.581
Constitutional symptoms^2^; % yes (n)	81.8 (18)	68.4 (13)	0.319
≥ 3 days of GC at time of FDG-PET/CT; % (n)	34.8 (8)	0.0	< 0.001
*Days of GC use; median (IQR)*	*5.0 (3.8–7.3)*		
≥ 3 days of GC at time of MRI; % (n)	47.8 (11)	0.0	< 0.001
*Days of GC use; median (IQR)*	*5.5 (4.8–7.0)*		
**B**	**GCA (*n* = 17)**	**No GCA (*n* = 15)**	**P-value**
Mean age in years (SD)	73.2 (5.2)	67.6 (8.0)	0.023
Female; % (n)	47.1 (8)	80.0 (12)	0.055
CRP (mg/L); median (IQR)	24.0 (7.3–112.8)	12.0 (6.0–27.0)	0.148
ESR (mm/hr); median (IQR)	52.0 (26.0–90.5)	27.0 (16.0–49.0)	0.038
Cranial symptoms^1^; % yes (n)	100.0 (17)	86.7 (13)	0.212
Constitutional symptoms^2^; % yes (n)	81.3 (13)	66.7 (10)	0.433

Patient characteristics of the A) total study population (*n* = 42) and B) study population used for analyses with CDUS (*n* = 32) after exclusion of patients that were deemed not reliably assessable by the experts. ^1^Cranial symptoms: headache, jaw claudication, visual loss and/or scalp tenderness. ^2^Constitutional symptoms: fatigue, weight loss and/or fever; GC = glucocorticoid, 40–60 mg/day; no patients had GCs at time of CDUS

### Inter- and intraobserver agreement of vessel assessment

**Fig. 1:**
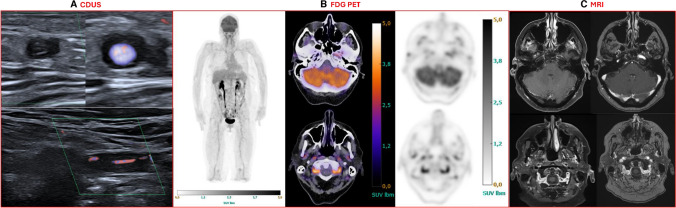
Example images of a GCA patient. A. CDUS images of the right temporal artery (transverse view, upper images) and left vertebral artery (longitudinal view, lower image). A halo sign may be observed in both arteries. B. FDG PET images with from left to right maximum intensity projection (MIP), fused PET/CT images of the head, and native PET images of the head. MIP image shows increased uptake in the common carotid arteries (grade 3 against liver) and the images of the head show increased uptake in the common superficial temporal and occipital arteries (grade 1 against background, upper images) and the vertebral and external carotid arteries (grade 2 against background, lower images). Grade 2 and 3 were considered positive for GCA. C. MRI images of the head with contrast enhanced T1-weighted MRI on the left and time-of-flight MRA on the right. Vessel wall thickening was observed in this patient in the common superficial temporal arteries and occipital arteries (both grade 3), most clearly visible here on the left. The lower image shows vessel wall thickening of the vertebral arteries (grade 2)

An overview of the inter-observer agreement for all assessable arteries on each imaging modality is shown in Table 2. Table 2 shows a subset of arteries that showed both inter- and intraobserver agreement (Cohen’s kappa > 0.00) for at least one of the imaging modalities. When vessels had interobserver agreement but intraobserver agreement could not be calculated (as only a subset of duplicated images was used), it was included for further analyses. The arteries not included in Table 2 were not considered for further analyses. Supplementary Table S1 shows inter- and intraobserver agreement for all assessed arteries. Example images are shown in Fig. 1.

**Table 2:** Inter-observer agreement for positivity of the assessed arteries on CDUS, FDG-PET/CT, and MRI

Artery	CDUS Cohen’s kappa (CI^95^)	FDG PET Cohen’s kappa (CI^95^)	MRI Cohen’s kappa (CI^95^)
Common Superficial Temporal Arteries	0.71 (0.51–0.90)	0.60 (0.27–0.91)	0.72 (0.54–0.90)
Frontal Superficial Temporal Arteries	0.78 (0.60–0.96)	0.71 (0.47–0.95)	0.74 (0.57–0.91)
Parietal Superficial Temporal Arteries	0.71 (0.51–0.92)	0.64 (0.32–0.97)	0.76 (0.57–0.94)
Posterior Deep Temporal Arteries	Not imaged	1.00	N/A
Anterior Deep Temporal Arteries	Not imaged	0.56 (0.12–1.00)	N/A
Maxillary Arteries	Not imaged	0.75 (0.60–0.91)	0.12 (−0.13–0.38)
Occipital Arteries	N/A	0.79 (0.50–1.00)	0.62 (0.36–0.88)
External Carotid Arteries	Not imaged	0.53 (0.21–0.86)	0.48 (0.05–0.90)
Vertebral Arteries	1.00	0.56 (0.35–0.76)	0.70 (0.50–0.89)
Common Carotid Arteries	0.00	0.60 (0.31–0.86)	Not imaged
Axillary Arteries	0.80 (0.57–1.00)	0.60 (0.39–0.80)	Not imaged
Subclavian Arteries	1.00	0.53 (0.32–0.74)	Not imaged
Innominate Artery	Not imaged	0.36 (−0.20–0.93)	Not imaged
Ascending Aorta	Not imaged	1.00	Not imaged
Aortic Arch	Not imaged	0.84 (0.55–1.00)	Not imaged
Descending Aorta	Not imaged	0.77 (0.47–1.00)	Not imaged
Abdominal Aorta	Not imaged	0.63 (0.36–0.92)	Not imaged

Arteries that were not imaged (CDUS) or not in the field of view (MRI) are shown if assessed on another modality. CDUS images of the occipital arteries were only saved in two patients and the deep temporal arteries could not be reliably assessed on MRI

#### CDUS

All patients (*N* = 32) had images of the superficial temporal arteries. Images of the axillary arteries were missing in one patient. Imaging of the subclavian arteries was performed in 10 patients, of the facial arteries in 13 patients, vertebral arteries in 4 patients, common carotid arteries in 16 patients, and occipital arteries in 2 patients. Inter and intra-observer agreement for the CDUS arteries were analyzed using Cohen’s kappa (Suppl. Table S1 A). Interobserver agreement was perfect for the subclavian and vertebral arteries, substantial for the axillary arteries and all segments of the superficial temporal arteries, and fair for the facial arteries. There was no agreement for the occipital and common carotid arteries, explaining why it is not included in Table 2. Intraobserver agreement was substantial for expert 1 and perfect for expert 2 considering the axillary arteries. This was almost perfect for the superficial temporal arteries for both experts. There was no intra-observer agreement in the facial arteries (therefore not included in Table 2). The subclavian, common carotid, and occipital arteries were not assessed positive for GCA in any of the duplicated scans. Therefore, only the superficial temporal (all branches), vertebral, axillary and subclavian arteries were included in Table 2 for CDUS.

#### FDG-PET/CT

Cohen’s kappa for inter and intraobserver agreement of FDG-PET/CT are shown in Supplementary Table S1 B. Interobserver agreement was substantial to perfect for the occipital, maxillary, and posterior deep temporal arteries. Interobserver agreement was fair in the innominate artery and no agreement (worse than chance) was observed for the internal carotid and facial arteries. Both experts were not able to assess FDG uptake in the ophthalmic arteries. All other assessed arteries (superficial temporal, anterior deep temporal, posterior auricular, external carotid, vertebral, axillary, common carotid, aorta) had inter-observer agreement and were therefore included in Table 2. The aorta, maxillary, superficial temporal (all segments), and vertebral arteries had intra-observer agreement for both experts. For the subclavian, axillary, and occipital arteries, one expert showed no agreement whereas the other expert did. No duplicated scans were positive for GCA in the innominate, common carotids, or posterior deep temporal arteries. There was no agreement for the internal carotid, posterior auricular and facial arteries (therefore excluded in Table 2). Therefore, the posterior deep temporal, anterior deep temporal, maxillary, occipital, external carotid, common carotid, and innominate arteries as well as the the aorta were included in Table 2 in addition to the arteries already included for CDUS.

#### MRI

Cohen’s kappa for inter- and intraobserver agreement of MRI are shown in Supplementary Table S1 C. The facial artery was never in the field of view of the MRI, the maxillary artery was not in the field of view of the MRI twice, and the vertebral artery was not in the field of view in one MRI scan. The ophthalmic, posterior auricular, anterior deep temporal, and posterior deep temporal arteries were not deemed adequately assessable by both experts as these were often not clearly visible. Interobserver agreement was substantial in the superficial temporal (all branches), occipital, and vertebral arteries. Of note, the maxillary artery had only slight interobserver agreement. Intraobserver agreement was slight to fair for the superficial temporal, maxillary, occipital, and vertebral arteries. However, for one expert there was no agreement in vertebral, posterior superficial temporal, maxillary, and occipital arteries. All arteries deemed assessable and reliable on MRI were already included in Table 2.

### Overall interobserver agreement for GCA diagnosis

Cohen’s kappa was calculated to assess interobserver agreement for the two experts per imaging modality. When one positive artery was used as a criterion for GCA diagnosis, interobserver agreement was almost perfect (0.88, 95% CI 0.61–1.00) for CDUS, moderate (0.54; 0.31–0.77) for FDG-PET/CT, and good (0.66; 0.44–0.88) for MRI. When using more than one positive artery as a criterion for diagnosis, CDUS agreement for the two experts on final diagnosis decreased to 0.81 (0.61–1.00) but FDG-PET/CT and MRI agreement respectively increased to 0.67 (0.45–0.89) and 0.80 (0.62–0.98).

### Overall diagnostic performance for CDUS, FDG-PET/CT and MRI

Sensitivity and specificity of CDUS, FDG-PET/CT and MRI with the diagnosis after 6 months made by the expert panel as a reference standard is described in Table 3. When one positive artery was used as a criterion for GCA diagnosis, sensitivity and specificity were respectively 82% (95% CI 59–95) and 93% (70–100) for CDUS (*n* = 32), 83% (63–93) and 84% (62–94) for FDG-PET/CT (*n* = 42) and 70% (49–84) and 89% (69–98) for MRI (*n* = 42). When multiple arteries were required to be positive for a diagnosis of GCA there was no change in diagnostic performance for CDUS, sensitivities for FDG-PET/CT and MRI respectively decreased to 78% (95% CI 58–90) and 65% (45–81), and specificities for FDG-PET/CT and MRI respectively increased to 100% (83–100) and 95% (75–100).

**Table 3:** Cross tabulation of the diagnostic performance of CDUS, FDG PET, and MRI assessments

			Expert 1		Expert 2		Consensus		
			GCA +	GCA-	GCA +	GCA-	GCA +	GCA-	*Se/Sp (CI*^*95*^*)*
CDUS	**Any**	+	13	2	14	1	14	1	82% (59–94%)
		-	4	13	3	14	3	14	93% (70–100%)
	**Any (> 1)**	+	11	1	14	1	14	1	82% (59–94%)
		-	6	14	3	14	3	14	93% (70–100%)
FDG-PET/CT	**Any**	+	19	5	16	0	19	3	83% (63–93%)
		-	4	14	7	19	4	16	84% (62–94%)
	**Any (> 1)**	+	19	2	16	0	18	0	78% (58–90%)
		-	4	17	7	19	5	19	100% (83–100%)
MRI	**Any**	+	13	2	18	2	16	2	70% (49–84%)
		-	10	17	5	17	7	17	89% (69–98%)
	**Any (> 1)**	+	12	2	16	2	15	1	65% (45–81%)
		-	11	17	7	17	8	18	95% (75–100%)

An imaging assessment was regarded as positive if any of the assessed arteries was positive (upper rows) or when more than 1 artery was positive (> 1, lower rows). Only previously determined reliably assessable arteries were used to calculate diagnostic performance. Cross-tabulations are shown for the individual experts and for the consensus score. Using the latter, the sensitivity (Se) and specificity (Sp) with 95% confidence interval (CI^95^) are shown

### Diagnostic performance of individual vessels

The diagnostic performance of each artery was determined to investigate which arteries are most relevant to include in imaging assessment. Sensitivity and specificity were calculated for the arteries that were previously determined to be reliably assessable (see Table 2) using the consensus score. Sensitivity and specificity with 95% confidence intervals for all graded individual arteries and grades per expert are shown in Supplementary Table S2.

#### CDUS

Sensitivity and specificity were highest in the superficial temporal arteries (76% and 93%, respectively) and sensitivity was lower in the axillary arteries with similar specificity (24% and 93%, respectively) for CDUS (Suppl. Table S2A). Fewer arteries were assessable for the vertebral arteries and the subclavian arteries. Of all patients with positive CDUS assessment in any artery, branches of the superficial temporal artery were CDUS positive in all GCA patients except one, in whom the axillary artery was positive.

#### FDG-PET/CT

For FDG-PET/CT, the highest sensitivity for increased uptake was in the vertebral and the maxillary arteries (61% and 52%), with 100% specificity for both. When only considering assessment of the maxillary and vertebral arteries on FDG-PET/CT, sensitivity for final GCA diagnosis was 70% (95% CI 49–84%) and specificity was 100% (95% CI 82–100%). The superficial temporal arteries had a sensitivity of 26% with a specificity of 89%, and the axillary arteries had a sensitivity of 22% with a specificity of 100%. Only one patient only had increased FDG uptake in the superficial temporal arteries and one other patient had isolated large vessel involvement on FDG-PET/CT. Other arteries on FDG-PET/CT had lower sensitivities. One patient without GCA had false positive increased FDG uptake in the aorta. Two patients had false positive increased FDG uptake in the superficial temporal artery. Increased FDG uptake in the other cranial arteries was 100% specific (Suppl. Table S2B). Two GCA patients had isolated increased uptake of the vertebral artery.

#### MRI

For MRI, vessel wall thickening in the superficial temporal artery showed highest sensitivity (60%) and vessel wall thickening in the maxillary, vertebral, and occipital artery was around 30% sensitive, but only the occipital artery was assessable in all patients. Vessel wall thickening was 100% specific in all assessed arteries except for the vertebral arteries (specificity of 89%) (Suppl. Table S2C). When considering only the superficial temporal and occipital arteries on MRI, sensitivity for final GCA diagnosis was 66% (45–82%) and specificity was 100% (82–100%).

### Overlap between imaging modalities

To determine the overlap in vascular assessment between CDUS, FDG-PET/CT and MRI, agreement was calculated using Cohen’s kappa (agreement between two imaging modalities) and Fleiss kappa (agreement between all three imaging modalities). This agreement score was calculated using the bilateral consensus scores of the two experts for each artery. Results are presented in Table 4. Agreement between CDUS and FDG-PET/CT was substantial to perfect for the axillary and subclavian artery (0.70 and 1.00, respectively). For the superficial temporal artery, agreement between CDUS with FDG-PET/CT was fair (0.28; Table 4A) and agreement with MRI was substantial (0.73; Table 4B). Agreement between FDG-PET/CT and MRI on the superficial temporal artery was moderate (0.40; Table 4C). The superficial temporal artery was the only artery reliably assessable on all three imaging modalities. Of the total number superficial temporal arteries assessed, 33% were positive on all three imaging modalities, corresponding with a Fleiss kappa of 0.76 (95% CI 0.62–0.91). When assessing individual branches, this was 5% for the common superficial temporal artery and 7% for both the frontal superficial temporal and parietal superficial temporal arteries, with Fleiss kappa of 0.34 (0.20–0.49), 0.37 (0.22–0.52) and 0.36 (0.20–0.52), respectively.

**Table 4.**
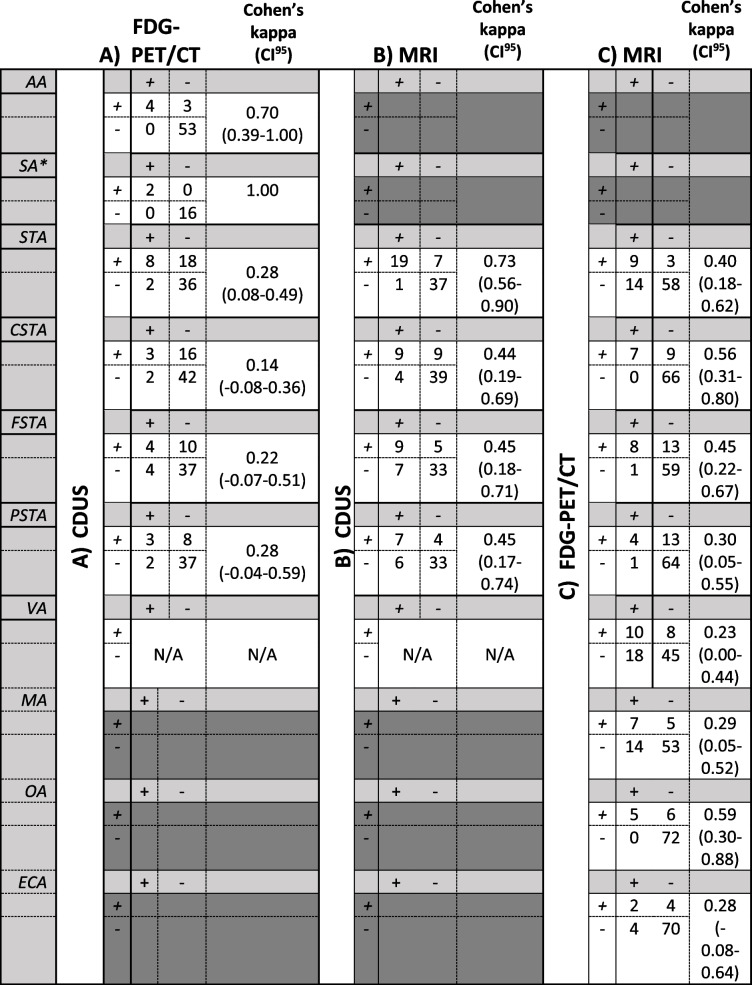
Cross tabulation of the CDUS, FDG-PET/CT and MRI assessments for bilateral arteries (reliably) assessable for at least 2 of the imaging modalities, using the consensus score of the two experts. Total numbers vary as not all arteries were assessable in every patient on multiple modalities. Comparisons with CDUS was performed in 32 patients. Cohen’s kappa and its 95%-confidence interval (CI95) are shown in the right column. A) CDUS comparison with FDG-PET/CT for the axillary artery and the branches of the superficial temporal artery. No patients were CDUS and FDG-PET/CT negative in the vertebral arteries, which precluded calculation of kappa statistic. B) CDUS comparison with MRI for the branches of the superficial temporal and the vertebral artery. No patients were CDUS and MRI negative in the vertebral arteries, which precluded calculation of kappa statistic. C) FDG-PET/CT comparison with MRI for the branches of the superficial temporal, the maxillary, occipital and external carotid artery

## Discussion

This is the first prospective study to describe and directly compare diagnostic performance and inter- and intraobserver agreement of vascular wall lesions on CDUS, FDG-PET/CT and MRI in patients with suspected GCA. This study evaluates the inter- and intraobserver agreement and sensitivity and specificity of individual arteries per imaging modality, evaluating reliability and validity of different vascular wall lesions. For CDUS, the temporal and axillary artery had the best inter- and intraobserver agreement with good sensitivity and specificity. For FDG-PET/CT, the vertebral, maxillary and axillary artery showed the best sensitivity and specificity with substantial agreement between the two experts, while for MRI this was the superficial temporal artery.

Following EULAR recommendations, CDUS of the temporal and axillary artery should be performed as a first test for GCA diagnosis [7]. Temporal and axillary artery CDUS was reproducible with good inter- and intraobserver agreement [24]. Every GCA positive CDUS had either a positive temporal or axillary artery, supporting that CDUS of the temporal and axillary artery should be performed in routine care [7]. A clear advantage of whole-body FDG-PET/CT is the ability to assess larger extracranial vessels, and our study shows good agreement between experts. Nevertheless, it is important to consider the low number of patients with FDG uptake higher than liver in the large vessels. One reason may be increased FDG uptake in the liver due to concurrent GC therapy and therefore decreasing sensitivity, an effect explained in a previous study. Additionally, one may also consider FDG uptake similar to liver as positive [17] and thus increase its sensitivity, but extensive analysis of FDG-PET/CT large vessel assessment was outside the scope of this study.

Axillary artery agreement between CDUS and FDG-PET/CT is high, and this finding is supported by previous literature [25]. A clear advantage of MRI is assessment of multiple cranial arteries [19]. Per scanning protocol the extracranial vessels are not scanned [18]. Interestingly, the maxillary and occipital artery were reliably assessable on both MRI and FDG-PET/CT. Regarding this, the maxillary artery had a better sensitivity, specificity and inter- and intraobserver agreement on FDG-PET/CT, while the occipital artery had similar interobserver agreement and better diagnostic performance on MRI. Hence, MRI has an advantage for reliable imaging of the occipital artery compared to the other two modalities. The additional value of this in GCA diagnosis should be further explored, but did not add in diagnosis in our small pilot population. The carotid, subclavian and innominate arteries and the aorta had lower sensitivity for GCA diagnosis, indicating that evaluation of these arteries alone does not discriminate GCA patients from non-GCA patients. A possible explanation is that they were less commonly affected in our patient population. Nevertheless, assessment of these arteries with an appropriate imaging modality (see Supplementary Table S1 and S2) could still aid in GCA diagnosis.

The superficial temporal artery could be assessed with good agreement between all three imaging modalities. Nevertheless, agreement of the temporal artery between FDG-PET/CT and CDUS or MRI was only fair, while there was better agreement between MRI and CDUS. In literature, it is described that FDG-PET/CT can accurately assess cranial arteries [14, 21]. In this study, assessment of the superficial temporal artery on FDG-PET/CT had good inter- and intraobserver agreement but had a low sensitivity of 26%. This can potentially be explained by the type of PET scanner used in our general hospital. Our results can be translated to other clinics without access to the newest generation of digital PET scanners, and suggest that caution should be taken when evaluating the temporal artery on FDG-PET/CT.

When comparing overall sensitivity and specificity of the three imaging modalities with previous literature, we observe comparable diagnostic performance between the three imaging modalities with high specificity [8, 9, 18, 26]. Specificity and interobserver agreement improved when two or more arteries were required to be positive for GCA diagnosis for FDG-PET/CT and MRI while this remained similar for CDUS. Interestingly, sensitivity was higher for all imaging modalities compared to the original pilot study, where imaging was assessed for the purpose of patient care. Considering the small population and overlapping confidence intervals, this should be carefully interpreted. Also, albeit still high, specificity was slightly lower as this was perfect in the original study, suggesting a more cautious interpretation in the clinic with access to patient information, time pressure and the ability for further testing. The sensitivity of FDG-PET/CT compared to the original pilot study was markedly increased and may be explained by the additional expert (RHJAS) who is an expert in FDG-PET/CT head-and-neck assessment of GCA and was not present in the previous study. If this is indeed the case, it highlights the need for training and expertise in head-and-neck assessment of GCA. Of note, on individual artery level, MRI interobserver agreement was low for mainly the maxillary artery, but intraobserver agreement was low for all arteries. The use of MRI as a diagnostic imaging modality in GCA is less well-established compared to FDG-PET/CT. As interobserver agreement was higher than intraobserver agreement, a learning curve should be considered especially considering the fact that the duplicated scans were scored last. More literature and more experience on MRI assessment in GCA may lead to higher intraobserver agreement [7].

A major strength of this study is the direct comparison of vascular wall inflammation in GCA suspected patients visualized on three imaging modalities, which provides a unique set of data. Furthermore, experts were blinded for patient characteristics when assessing the scans, ensuring unbiased evaluation. The use of patients with suspected GCA without final diagnosis of GCA provides a clinically relevant control group. For analyses considering diagnostic performance, arteries that showed no inter- and/or intraobserver agreement were excluded, as reproducibility is necessary for reliable diagnostic testing. This study has some limitations. First, a small number of patients is used as this was a sensitivity analysis of a pilot study, resulting in large confidence intervals for Kappa values. Also, we chose to include a limited number of duplicated scans in our study. It was not logistically feasible to increase this number. Nevertheless, the small number of duplicated scans does give some insight in intra-observer agreement, which has not been studied in this context before. For CDUS, the temporal and the axillary artery were assessed for standard diagnostic work-up, however the other arteries were not systematically assessed. This led to a small number of assessable arteries, warranting careful interpretation of inter- and intraobserver agreement and diagnostic performance for individual arteries. However, we believe our prospective study design provides a good first evaluation of vascular wall lesions by the three imaging modalities. Furthermore, the CDUS patients excluded were the first consecutive patients included in the original pilot study as the CDUS machine was replaced, therefore there was no selection bias for higher-quality CDUS. In the original pilot study, point-of-care CDUS was performed for diagnostic purposes. Using static images for re-assessment eliminated dynamical scanning of the artery, which was done to assess the presence of GCA for clinical practice. Furthermore, GC treatment could have influenced FDG-PET/CT and MRI results, however imaging was performed as quickly as possible after diagnostic work-up. This reflects daily clinical practice, as imaging modalities are not always immediately available. Lastly, the reference standard used to diagnose GCA could have influenced diagnostic performance as the lack of gold standard remains an issue in assessing diagnostic performance in GCA and our used reference standard could be inherent to actions of the treating physician.

In conclusion, based on assessment of vascular wall lesions visualized on CDUS, FDG-PET/CT and MRI by two experts blinded for patient characteristics, this study demonstrated that the temporal artery can reliably be assessed by CDUS and MRI, and the axillary artery by CDUS and FDG-PET/CT with high agreement between the two modalities. This supports EULAR recommendations to perform CDUS of the temporal and axillary arteries as a first test. In addition, the vertebral and maxillary artery can be assessed by FDG-PET/CT with good validity, even though moderate reliability of the vertebral artery should be taken into account. Low intraobserver agreement for MRI assessments should be considered. The occipital, external carotid, common carotid, subclavian and innominate arteries and the aorta can still be reliably assessed by an appropriate imaging modality and can thus contribute to diagnosis when involvement is suspected.

## Supplementary Information

Below is the link to the electronic supplementary material.

Supplementary file1 (DOCX 59 KB)

## Data Availability

The datasets generated during and/or analysed during the current study are available from the corresponding author on reasonable request.
