# Supplementary material for: An in-depth comparison of vascular inflammation on ultrasound, FDG-PET/CT and MRI in patients with suspected giant cell arteritis
Source: Eur J Nucl Med Mol Imaging. 2025 Feb 4;52(7):2491–501. doi: 10.1007/s00259-025-07088-3 (PMC12119766; doi:10.1007/s00259-025-07088-3)
Supplement: Supplementary file 1 — Supplementary file1 (DOCX 59 KB) [file 259_2025_7088_MOESM1_ESM.docx]

|  | **Artery** | **Inter-observer agreement** | **Intra-observer agreement #1** | **Intra-observer agreement #2** |
| --- | --- | --- | --- | --- |
| **A)**  **CDUS** | **Common Superficial Temporal Artery** | 0.705 (0.509 – 0.902) | 0.800 (0.432 – 1.000) | 0.800 (0.432 – 1.000) |
|  | **Frontal Superficial Temporal Artery** | 0.781 (0.601 – 0.960) | 1.000 | 1.000 |
|  | **Parietal Superficial Temporal Artery** | 0.714 (0.507 – 0.920) | 0.800 (0.436 – 1.000) | 0.800 (0.436 – 1.000) |
|  | **Occipital Artery** | 0.000 | N/A* | N/A* |
|  | **Facial Artery** | 0.222 (-0.197 – 0.641) | -0.174 (-0.423 – 0.076) | 0.000 |
|  | **Vertebral Artery** | 1.000 | N/A* | N/A* |
|  | **Common Carotid Artery** | 0.000 | N/A* | N/A* |
|  | **Axillary Artery** | 0.795 (0.571 – 1.000) | 0.625 (-0.027 – 1.000) | 1.000 |
|  | **Subclavian Artery** | 1.000 | N/A* | N/A* |
| **B)**  **FDG-PET/CT** | **Common Superficial Temporal Artery** | 0.60 (0.27-0.91) | 0.60 (0.13-1.00) | 1.00 |
|  | **Frontal Superficial Temporal Artery** | 0.64 (0.32-0.97) | 0.82 (0.48-1.00) | 0.77 (0.33-1.00) |
|  | **Parietal Superficial Temporal Artery** | 0.71 (0.47-0.95) | 1.00 | 1.00 |
|  | **Posterior Deep Temporal Artery** | 1.00 | N/A* | N/A* |
|  | **Anterior Deep Temporal Artery** | 0.56 (0.12-1.00) | -0.11 (-0.30-0.07) | N/A* |
|  | **Maxillary Artery** | 0.75 (0.60-0.91) | 0.48 (0.02-0.93) | 0.39 (-0.03-0.80) |
|  | **Posterior Auricular Artery** | 0.32 (-0.06-0.70) | 0.00 | 0.00 |
|  | **Occipital Artery** | 0.79 (0.50-1.00) | 1.00 | 0.00 |
|  | **Facial Artery** | -0.03 (-0.05-0.00) | -0.14 (-0.28—0.00) | N/A* |
|  | **Ophthalmic Artery** | N/A | N/A* | N/A* |
|  | **External Carotid Artery** | 0.53 (0.21-0.86) | 0.60 (0.13-1.00) | 0.00 |
|  | **Internal Carotid Artery** | 0.00 | 0.00 | 0.00 |
|  | **Vertebral Artery** | 0.56 (0.35-0.76) | 0.47 (0.02-0.91) | 0.71 (0.36-1.00) |
|  | **Common Carotid Artery** | 0.60 (0.31-0.86) | N/A* | N/A* |
|  | **Axillary Artery** | 0.60 (0.39-0.80) | 0.39 (-0.03-0.80) | 0.00 |
|  | **Subclavian Artery** | 0.53 (0.32-0.74) | 0.31 (-0.05-0.67) | 0.00 |
|  | **Innominate Artery** | 0.36 (-0.20-0.93) | N/A* | N/A* |
|  | **Ascending Aorta** | 1.00 | 1.00 | 1.00 |
|  | **Aortic Arch** | 0.84 (0.55-1.00) | 0.00 (0.00-0.00) | 1.00 |
|  | **Descending Aorta** | 0.77 (0.47-1.00) | 0.34 (-0.07-1.00) | 1.00 |
|  | **Abdominal Aorta** | 0.63 (0.36-0.92) | 0.34 (-0.07-1.00) | 0.39 (-0.20-0.97) |
| **C)**  **MRI** | **Common Superficial Temporal Artery** | 0.72 (0.54-0.90) | 0.09 (-0.42-0.61) | 0.33 (-0.19-0.86) |
|  | **Frontal Superficial Temporal Artery** | 0.74 (0.57-0.91) | 0.33 (-0.20-0.86) | 0.33 (-0.19-0.86) |
|  | **Parietal Superficial Temporal Artery** | 0.76 (0.57-0.94) | 0.09 (-0.42-0.61) | -0.20 (-0.40-0.00) |
|  | **Posterior Deep Temporal Artery** | N/A* | N/A* | N/A* |
|  | **Anterior Deep Temporal Artery** | N/A* | N/A* | N/A* |
|  | **Maxillary Artery** | 0.12 (-0.13-0.38) | -0.24 (-0.48-0.00) | 0.09 (-0.42-0.61) |
|  | **Posterior Auricular Artery** | N/A* | N/A* | N/A* |
|  | **Occipital Artery** | 0.62 (0.36-0.88) | -0.14 (-0.28—0.00) | 0.09 (-0.42-0.61) |
|  | **Facial Artery** | N/A* | N/A* | N/A* |
|  | **Ophthalmic Artery** | N/A* | N/A* | N/A* |
|  | **External Carotid Artery** | 0.48 (0.05-0.90) | 0.00 | N/A* |
|  | **Internal Carotid Artery** | N/A* | N/A* | N/A* |
|  | **Vertebral Artery** | 0.70 (0.50-0.89) | 0.24 (-0.03-0.52) | -0.04 (-0.47-0.39) |

***Supplementary Table S1*** *Inter- and intraobserver agreement for* *all assessed arteries on CDUS, FDG-PET/CT and MRI; *not imaged or not assessable in the subset of duplicated images, which preluded the calculation of Cohen’s kappa; A) No GCA patients had an imaged positive common carotid artery.* *B) FDG uptake in the ophthalmic arteries could not be assessed by the two experts; C) The internal carotid arteries were never positive and the facial arteries were always outside the field of view. The ophthalmic, posterior auricular and deep temporal arteries could not be assessed by the two experts.*

| **Artery** |  | **Expert 1** | | **Expert 2** | | **Consensus** | | |
| --- | --- | --- | --- | --- | --- | --- | --- | --- |
|  |  | *GCA+* | *GCA-* | *GCA+* | *GCA-* | *GCA+* | *GCA-* | *Se/sp (CI^95^)* |
| **Any** | *US+* | 13 | 2 | 14 | 1 | 14 | 1 | *Se 82% (59-94%)*  *Sp 93% (70-100%)* |
|  | *US-* | 4 | 13 | 3 | 14 | 3 | 14 |  |
| **Any (>1)** | *US+* | 11 | 1 | 14 | 1 | 14 | 1 | *Se 82% (59-94%)*  *Sp 93% (70-100%)* |
|  | *US-* | 6 | 14 | 3 | 14 | 3 | 14 |  |
| **STA** | *US+* | 11 | 1 | 13 | 1 | 13 | 1 | *76% (52-90%)*  *93% (70-100%)* |
|  | *US-* | 6 | 14 | 4 | 14 | 4 | 14 |  |
| **VA*** | *US+* | 1 | 0 | 1 | 0 | 2 | 0 | *25% (4-59%)*  *N/A* |
|  | *US-* | 3 | 0 | 4 | 0 | 6 | 0 |  |
| **AA** | *US+* | 4 | 2 | 6 | 1 | 4 | 1 | *24% (10-47%)*  *93% (70-100%)* |
|  | *US-* | 12 | 13 | 10 | 14 | 13 | 14 |  |
| **SA*** | *US+* | 1 | 0 | 1 | 0 | 1 | 0 | *11% (0-44%)*  *100% (44-100%)* |
|  | *US-* | 6 | 3 | 6 | 3 | 8 | 3 |  |

**Supplementary Table S2A** Cross tabulation of positive and negative CDUS assessment for the individual arteries. The upper rows contain cross tabulations for any of the individual arteries being positive or more than one (>1) artery being positive, respectively. Sensitivity and specificity for the consensus scores are shown in the most right column. STA, superficial temporal arteries; VA, vertebral arteries; AA, axillary arteries; SA, subclavian arteries. No GCA- patients had images of the vertebral arteries. *Fewer total patients because cot all patients had images present for these arteries or the images were not always assessable.

|  |  | **Expert 1** | | **Expert 2** | | **Consensus** | | |
| --- | --- | --- | --- | --- | --- | --- | --- | --- |
|  |  | *GCA+* | *GCA-* | *GCA+* | *GCA-* | *GCA+* | *GCA-* | *Accuracy (CI^95^)* |
| **Any** | *PET+* | 19 | 5 | 16 | 0 | 19 | 3 | Se 83% (63-93%) |
|  | *PET-* | 4 | 14 | 7 | 19 | 4 | 16 | Sp 84% (62-94%) |
| **Any (>1)** | *PET+* | 19 | 2 | 16 | 0 | 18 | 0 | Se 78% (58-90%) |
|  | *PET-* | 4 | 17 | 7 | 19 | 5 | 19 | Sp 100% (83-100%) |
| **Head/neck arteries** | *PET+* | 16 | 5 | 14 | 0 | 17 | 2 | 73% (54-87%) |
|  | *PET-* | 7 | 14 | 9 | 19 | 6 | 17 | 89% (69-98%) |
| **STA** | *PET+* | 6 | 4 | 4 | 0 | 6 | 2 | 26% (13-46%) |
|  | *PET-* | 17 | 15 | 19 | 19 | 17 | 17 | 89% (69-98%) |
| **PDTA** | *PET+* | 1 | 0 | 1 | 0 | 1 | 0 | 4% (0-21%) |
|  | *PET-* | 22 | 19 | 22 | 19 | 22 | 19 | 100% (83-100%) |
| **ADTA** | *PET+* | 2 | 1 | 1 | 0 | 2 | 0 | 9% (2-27%) |
|  | *PET-* | 21 | 18 | 22 | 19 | 21 | 19 | 100% (83-100%) |
| **MA** | *PET+* | 11 | 0 | 12 | 0 | 12 | 0 | 52% (33-71%) |
|  | *PET-* | 12 | 19 | 11 | 19 | 11 | 19 | 100% (83-100%) |
| **ECA** | *PET+* | 4 | 0 | 3 | 0 | 4 | 0 | 17% (7-37%) |
|  | *PET-* | 19 | 19 | 20 | 19 | 19 | 19 | 100% (83-100%) |
| **VA** | *PET+* | 10 | 0 | 11 | 0 | 14 | 0 | 61% (41-78%) |
|  | *PET-* | 13 | 19 | 12 | 19 | 9 | 19 | 100% (83-100%) |
| **OA** | *PET+* | 3 | 0 | 2 | 0 | 3 | 0 | 13% (5-32%) |
|  | *PET-* | 20 | 19 | 21 | 19 | 20 | 19 | 100% (83-100%) |
| **Large vessels** | *PET+* | 6 | 1 | 5 | 0 | 5 | 1 | 22% (10-42%) |
|  | *PET-* | 17 | 18 | 18 | 19 | 18 | 18 | 95% (75-100%) |
| **CCA** | *PET+* | 2 | 0 | 2 | 0 | 2 | 0 | 9% (2-27%) |
|  | *PET-* | 21 | 19 | 21 | 19 | 21 | 19 | 100% (83-100%) |
| **AA** | *PET+* | 5 | 0 | 5 | 0 | 5 | 0 | 22% (10-42%) |
|  | *PET-* | 18 | 19 | 18 | 19 | 18 | 19 | 100% (83-100%) |
| **SA** | *PET+* | 6 | 0 | 4 | 0 | 4 | 0 | 17% (7-37%) |
|  | *PET-* | 17 | 19 | 19 | 19 | 19 | 19 | 100% (83-100%) |
| **IA** | *PET+* | 2 | 0 | 2 | 0 | 2 | 0 | 9% (2-27%) |
|  | *PET-* | 21 | 19 | 21 | 19 | 21 | 19 | 100% (83-100%) |
| **Aorta** | *PET+* | 3 | 1 | 4 | 0 | 3 | 1 | 13% (5-32%) |
|  | *PET-* | 20 | 18 | 19 | 19 | 20 | 18 | 95% (75-100%) |

**Supplementary Table S2B** Cross tabulation of positive and negative FDG PET assessment for the individual arteries. The graded cut-off for the large vessels was uptake higher than liver and the cut-off for the cranial arteries was higher than background. The upper rows contain cross tabulations for any of the individual arteries being positive or more than one (>1) artery being positive, respectively. The ‘head/neck arteriesl’ row considers only cranial arteries and the ‘Large Vessel’ row considers only large vessels. Sensitivity and specificity for the consensus scores are shown in the most right column. STA, superficial temporal arteries; PDTA, posterior deep temporal arteries; ADTA, anterior deep temporal arteries; MA, maxillary arteries; ECA, external carotid arteries; VA, vertebral arteries; OA, occipital arteries; AA, axillary arteries; SA, subclavian arteries; IA, innominate arteries. No GCA- patients had images of the vertebral arteries. *Fewer total patients because cot all patients had images present for these arteries or the images were not always assessable.

|  |  | **Expert 1** | | **Expert 2** | | **Consensus** | | |
| --- | --- | --- | --- | --- | --- | --- | --- | --- |
|  |  | *GCA+* | *GCA-* | *GCA+* | *GCA-* | *GCA+* | *GCA-* | *Se/sp (CI^95^)* |
| **Any** | *MRI+* | 13 | 2 | 18 | 2 | 16 | 2 | Se 70% (49-84%) |
|  | *MRI-* | 10 | 17 | 5 | 17 | 7 | 17 | Sp 89% (69-98%) |
| **Any (>1)** | *MRI+* | 12 | 2 | 16 | 2 | 15 | 1 | Se 65% (45-81%) |
|  | *MRI-* | 11 | 17 | 7 | 17 | 8 | 18 | Sp 95% (75-100%) |
| **STA** | *MRI+* | 11 | 1 | 14 | 1 | 14 | 0 | 60% (41-78%) |
|  | *MRI-* | 12 | 18 | 9 | 18 | 9 | 19 | 100% (83-100%) |
| **MA*** | *MRI+* | 7 | 0 | 6 | 2 | 7 | 0 | 33% (17-55%) |
|  | *MRI-* | 15 | 19 | 14 | 16 | 14 | 19 | 100% (83-100%) |
| **ECA*** | *MRI+* | 3 | 0 | 1 | 0 | 3 | 0 | 14% (5-35%) |
|  | *MRI-* | 20 | 19 | 18 | 17 | 18 | 19 | 100% (83%-100%) |
| **VA*** | *MRI+* | 7 | 2 | 10 | 1 | 8 | 2 | 36% (20-57%) |
|  | *MRI-* | 15 | 17 | 10 | 18 | 14 | 17 | 89% (69-98%) |
| **OA** | *MRI+* | 5 | 0 | 10 | 1 | 7 | 0 | 30% (16-51%) |
|  | *MRI-* | 18 | 19 | 13 | 18 | 16 | 19 | 100% (83-100%) |

**Supplementary Table S2C** Cross tabulation of positive and negative MRI assessment (cut-off grade >1) for the individual arteries. The upper rows contain cross tabulations for any of the individual arteries being positive or more than one (>1) artery being positive, respectively. Sensitivity and specificity for the consensus scores are shown in the most right column. STA, superficial temporal arteries; MA, maxillary arteries; ECA, external carotid arteries; VA, vertebral arteries; OA, occipital arteries.. *Fewer total patients because cot all patients had images present for these arteries or the images were not always assessable.
